# The Influence of COVID-19 Vaccination on Daily Cases, Hospitalization, and Death Rate in Tennessee, United States: Case Study

**DOI:** 10.2196/29324

**Published:** 2021-08-12

**Authors:** Ali Roghani

**Affiliations:** 1 Division of Epidemiology University of Utah Salt Lake City, UT United States

**Keywords:** COVID-19, pandemic, vaccination, vaccine, strategy, vaccination strategy, hospitalization, mortality rates, older adults, mortality

## Abstract

**Background:**

The COVID-19 outbreak highlights our vulnerability to novel infections, and vaccination remains a foreseeable method to return to normal life. However, infrastructure is inadequate for the immediate vaccination of the whole population. Therefore, policies have adopted a strategy to vaccinate older adults and vulnerable populations while delaying vaccination for others.

**Objective:**

This study aimed to understand how age-specific vaccination strategies reduce daily cases, hospitalizations, and death rates using official statistics for Tennessee, United States.

**Methods:**

This study used publicly available data on COVID-19, including vaccination rates, positive cases, hospitalizations, and deaths from the Tennessee Department of Health. Data from the first date of vaccination (December 17, 2020) to March 3, 2021, were retrieved. The rates were adjusted by 2019 data from the US Census Bureau, and age groups were stratified into 10-year intervals starting with 21 years of age.

**Results:**

The findings showed that vaccination strategy can reduce the numbers of patients with COVID-19 in all age groups, with lower hospitalization and death rates in older populations. Older adults had a 95% lower death rate from December to March; no change in the death rate of other age groups was observed. The hospitalization rate was reduced by 80% for people aged ≥80 years, while people who were 50 to 70 years old had nearly the same hospitalization rate as prior to vaccination.

**Conclusions:**

This research indicates that targeting older age groups for vaccination is the optimal way to avoid higher transmissions and reduce hospitalization and death rates.

## Introduction

In December 2019, the novel coronavirus SARS-CoV-2 in Wuhan, China, caused an outbreak of the disease COVID-19 [1]. In the next few weeks, COVID-19 became the main headline worldwide, and daily cases and deaths increased considerably [2]. By the end of November 2020, more than 14 million infections and 279,000 deaths had been confirmed nationally in the United States, making it the country with the highest number of cases in the world at that time [3]. With more than 368,000 daily cases and 4500 deaths, Tennessee has been identified as one of the hardest-hit states in the United States [3].

Vaccination and social distancing are essential factors for COVID-19 prevention [4]. COVID-19 vaccination rollout in Tennessee started on December 17, 2020, and by March 3, 2021, 13.3% of the population had already received an mRNA vaccine such as the Pfizer BNT162b2 (Tozinameran) and the Moderna mRNA-1273 [5,6]. In addition to reduced interpersonal contact and physical distancing, vaccination programs control virus spread and reduce the number of deaths [7]. While COVID-19 cases and deaths were highest in January 2021 in the United States, manufacturers currently cannot cover the enormous vaccination demand. As the vaccine supply is limited, it is crucial to prioritize who receives the vaccine; therefore, groups at the highest risk of getting the virus or individuals who are seriously ill have been prioritized for vaccination. Previous research has shown that prioritizing younger populations will significantly impact the reduction of COVID-19 cases relative to prioritizing older age groups. However, prioritizing younger age groups is associated with the lowest reduction in COVID-19 mortality compared to other approaches [8]. In addition, Tennesseans are eligible for vaccines based solely on their age, and these age-based phases have run simultaneously with those with high-risk health conditions.

The objective of this paper was to model how the vaccination program in Tennessee is likely to change COVID-19 daily cases, hospitalization, and deaths among adults.

## Methods

This study used publicly available data on COVID-19, including vaccination rates, positive cases, hospitalizations, and deaths from the Tennessee Department of Health at the state level [9]. The rates were also adjusted by 2019 data from the US Census Bureau [10].

### Measures

This study comprised data from the first date of vaccinations, December 17, 2020, to March 3, 2021. The Tennessee Department of Health provides data on the first and second doses of vaccinations, cases, hospitalization, and deaths daily. The data were stratified based on 10-year intervals starting from 21 years of age to ≥81 years.

### Statistical Analysis

The methodology for generating a descriptive time series of vaccinations, daily cases, hospitalizations, and deaths involved two steps. The first was to convert aggregate data to daily data to create time-series data. The second was to adjust the data for each age group by the census data, including the percentage of Tennesseans who received vaccines, had COVID-19, were hospitalized, and died. The goal was to produce a series of trends over consecutive time intervals to understand the changes in COVID-19 cases, hospitalizations, and deaths after the onset of vaccinations. The data were analyzed with the R programming language (version 3.5.2, R Core Team) [11].

## Results

**Figure 1:**
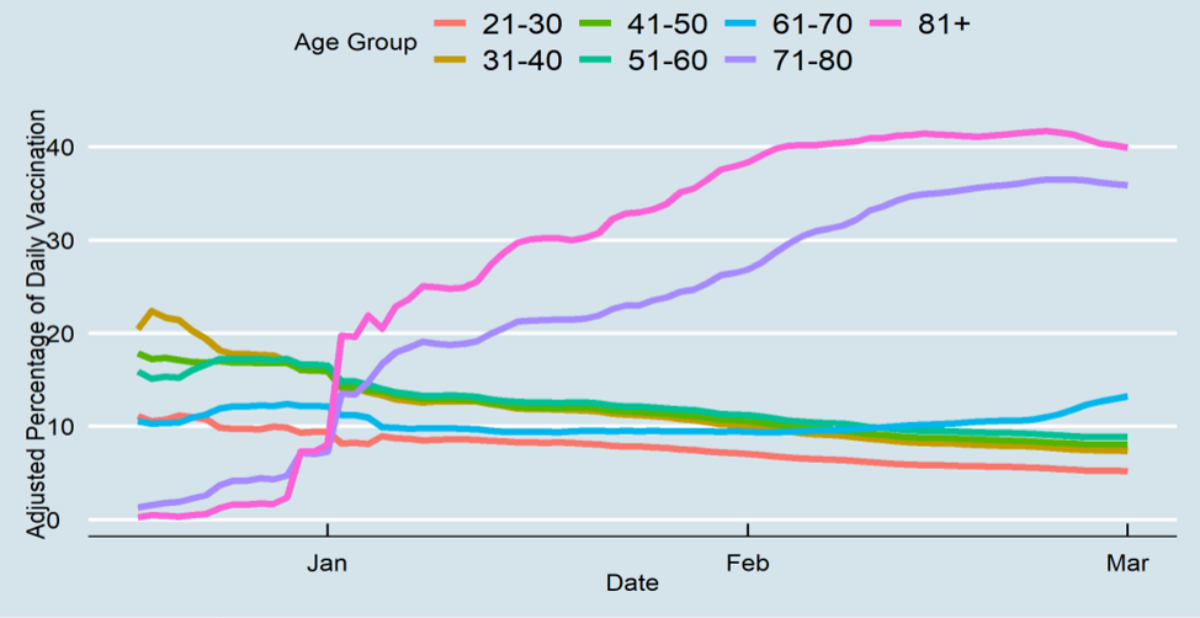
First-dose vaccine distribution of Tennesseans by age group.

**Figure 2:**
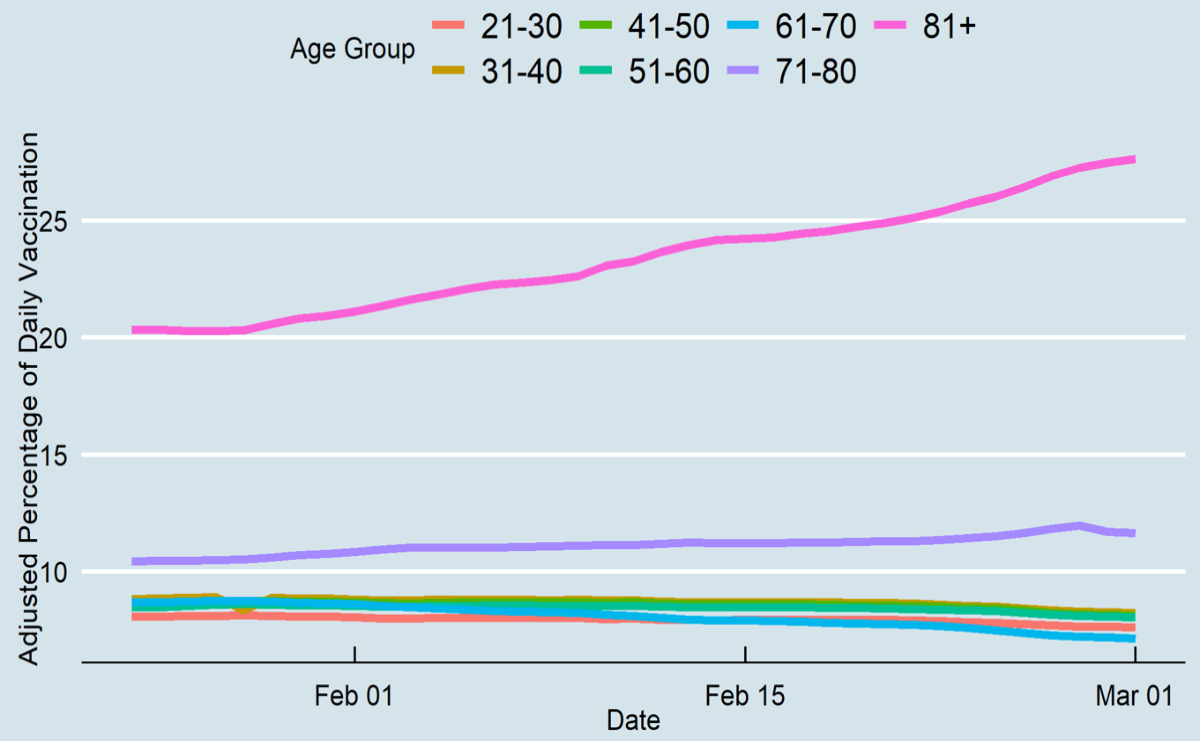
Daily fully vaccinated rates of Tennesseans by age group.

During the first 78 days of vaccination in Tennessee, 953,568 individuals received their first dose, and 495,032 individuals received their second dose. Of those vaccinated, 18.2% (n=173,549) and 30.3% (n=288,931) of vaccines were administered to people of aged ≥81 years and 71-80 years, respectively, which shows that nearly half of the vaccines were given to those older than 71 years. Figure 1 indicates the percentage of those who received their first vaccine from December 17, 2020, to March 3, 2021. Individuals who were under 70 years had a higher vaccination rate before January 2021; however, from January to March 3, 40% of people aged ≥81 years and 36% of those aged 71-80 years had been vaccinated, which shows that most of the vaccines were administered to these two age groups. Figure 2 shows that only 25% (n=63,202) of Tennesseans who were older than 81 years received their second vaccine, while a smaller percentage of other age groups were also vaccinated. Although those aged 71-80 years had an approximately equal rate of receiving the first vaccine dosage compared to those aged ≥81 years, they received their second dosage at a similar rate to other age groups.

**Figure 3:**
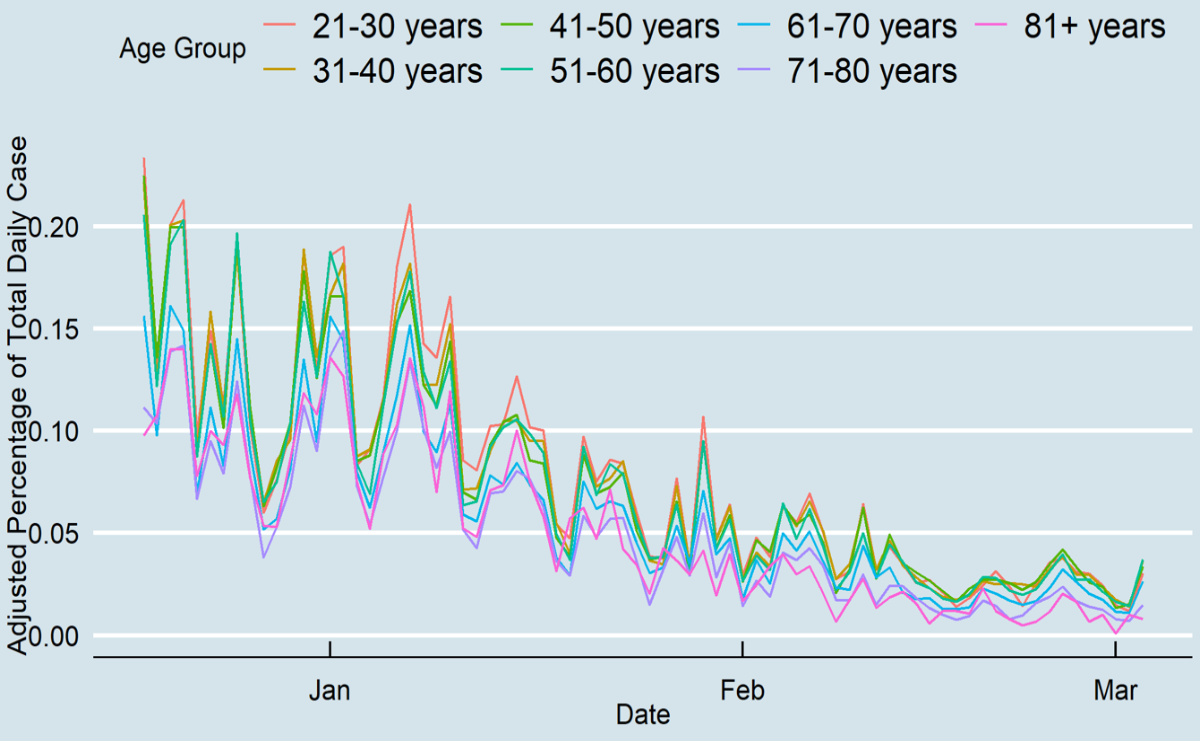
COVID-19 daily cases in Tennessee.

**Figure 4:**
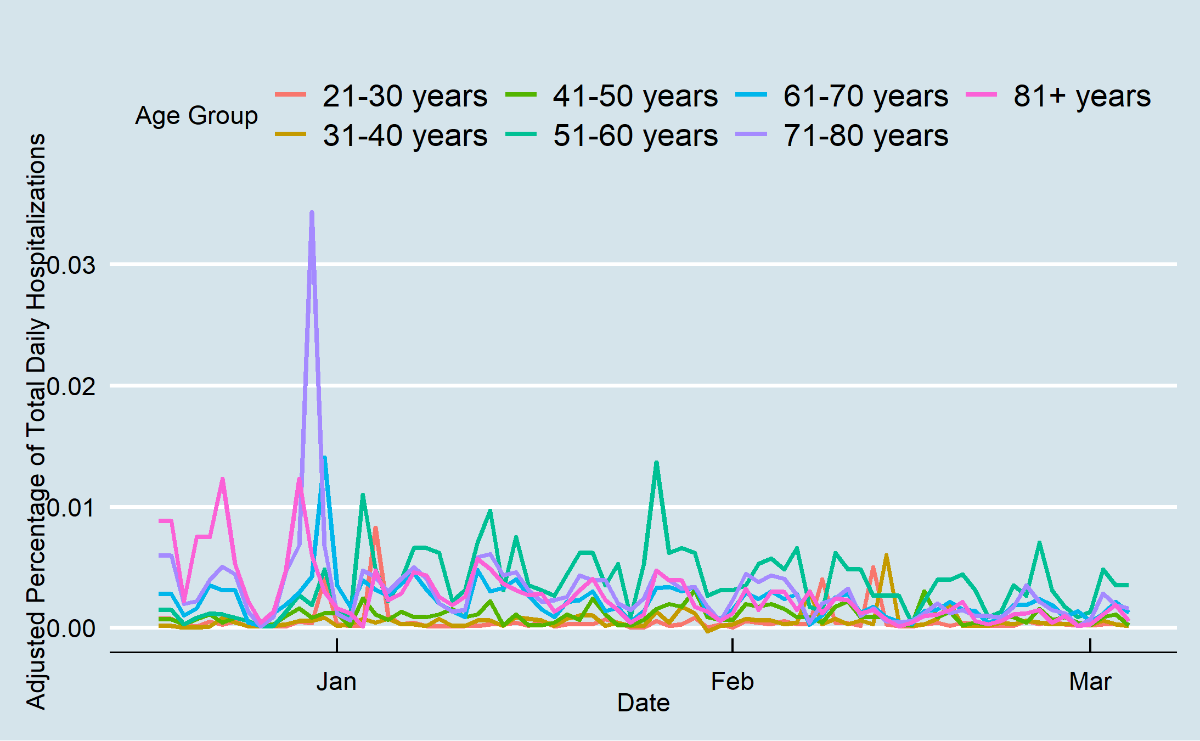
COVID-19 daily hospitalizations in Tennessee.

**Figure 5:**
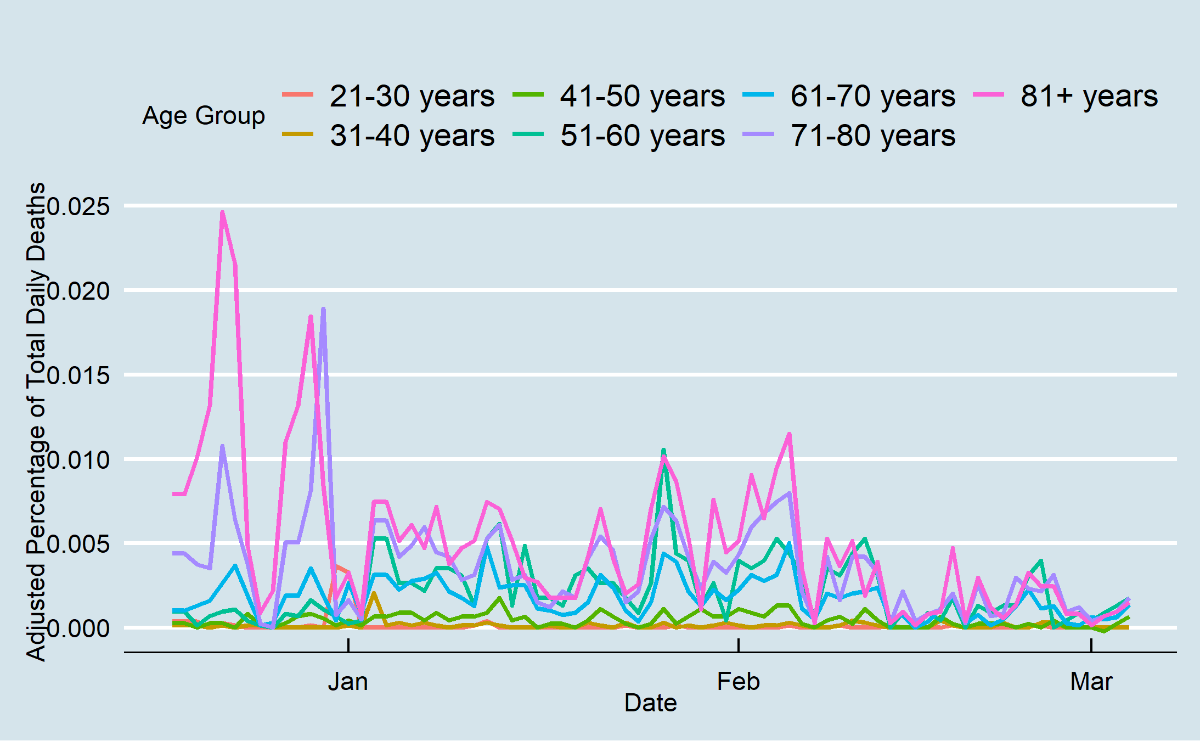
COVID-19 daily deaths in Tennessee.

Daily cases for all age groups decreased inevitably after the onset of vaccination to day 78 (Figure 3). Daily cases decreased for younger people from approximately 0.2% at the end of January to less than 0.05% at the end of the study period. The proportional changes were considerably higher for older adults during the study period (from 0.1% to nearly 0.01% of daily cases). Before vaccination, older age groups had the highest hospitalization rates (Figure 4). The rate decreased at the end of the study period from 0.01% of Tennessee’s older population to 0.003%. There was no substantial change in other age groups’ hospitalization rates, although the age groups of 51-60 years and 61-70 years had high hospitalization rates on some days. From mid-February, the ≥71 years age group did not experience high hospitalization rates, and the 51-60 years age group had nearly the highest daily hospitalization rate. Lastly, the death rates among those aged ≥71 years decreased, while there was no change in the death rates of other age groups during the study period. Although Tennesseans over 71 years accounted for 0.015% of the daily deaths pertaining to COVID-19 at the end of 2020, this percentage decreased substantially to 0.003% at the end of the study period (Figure 5). The results showed that the gap between older and younger adults was high prior to vaccination, and after vaccination, the differences diminished, which indicates that all age groups had a similar death rate.

## Discussion

### Principal Findings

COVID-19 continues to spread in the United States, and hospitalization and death rates remain high. Vaccines offer hope for better conditions, but an effective vaccination strategy is needed to stop the pandemic and restore people’s everyday lives. Unfortunately, vaccine doses are being delivered slowly and sporadically, which means it is difficult for most people to be vaccinated at this time, even if they are eligible. Based on the current policy, high-risk groups such as first responders, older adults, and individuals with high-risk health conditions should receive the vaccines first [12]. In this study, data from the onset of COVID-19 vaccination in Tennessee (December 17, 2020) was used to understand how age-specific vaccination strategies changed daily cases, hospitalization, and death rate. The figures indicate that phase 1 of the vaccination strategy reduced the number of patients in all age groups, with lower hospitalization and death rates for older adults. The finding demonstrates that more than half of the vaccines were administered to those greater than 70 years; this was a practical approach in blocking transmission in the older adult population and other age groups. COVID-19 daily cases in older groups decreased by 90% from the end of 2020 to the end of February 2021. In addition, less than half of the vaccines were used for those aged under 70 years; this group had 80% lower daily cases at the end of the study period compared to the vaccine initiation date. Although this study cannot confirm the association between the onset of vaccination and the considerable reduction in COVID-19 transmission among younger age groups, the data indicate a significant decrease in daily cases among Tennesseans in all age groups. Moreover, 25% of people who were older than 81 years received the vaccines, and around 10% of other age groups received the second dosage. However, the ≥81 years age group did not have better results than their counterparts aged 71-80 years in terms of hospitalizations and death rate. This study included 78 days of vaccination statistics; thus, it is too early to draw conclusions on the influence of the second dose. Future studies should be conducted over a longer period to obtain more accurate results concerning the second dose.

Vaccines led to substantial reductions in hospitalization and mortality among older age groups in Tennessee. People older than 80 years had a 95% reduced death rate compared to mid-December. The death rate of the 71-80 years age group decreased during the study period as well; however, the 61-70 years age group had almost the same death rate from mid-December to the end of February. The data showed that there was no change in death rate in other age groups. Hospitalization of Tennesseans aged greater than 80 years was reduced by 80% in the study period, while people between 50-70 years experienced nearly the same hospitalization rate. Indeed, individuals who were between 51-60 years of age had the highest hospitalization rates in Tennessee. Although the data cannot identify people with higher risk, the higher hospitalization rate among the younger population implies that the health system in Tennessee could not efficiently identify people at higher risk. A previous study showed that a significant proportion of people who had two or more chronic conditions simultaneously are more likely to be hospitalized due to SARS-CoV-2 infection [13]. Additionally, while health workers are placed at the highest risk groups, immunizing this population and supplying personal protective equipment will help increase the resiliency of the health system during the epidemic [14].

### Limitations

The findings should be considered in the context of several data limitations. Individual-level data was not used to estimate hospitalization risks, mortality rate, and COVID-19 transmissions. Moreover, several studies [15,16] indicate that racial and ethnic disparities in health systems increase the risk of getting sick, being hospitalized, and dying from COVID-19. Future studies should examine vaccination in different racial groups by age to estimate to prioritize vaccination. Additionally, the data do not include nonpharmaceutical public health control measures, which would be an essential way of controlling daily cases [17]. Although statistics on cases, hospitalizations, and deaths prior to the onset of vaccination could provide a more accurate picture regarding the changes due to vaccination, the preliminary analysis showed that the gaps between older and younger age groups were consistent before the onset of vaccination up to the end of January. Since February, however, the gap between older and younger age groups has diminished considerably. The reason why there were no immediate changes after vaccination uptake could be due to two factors. First, previous research has shown that it takes some time to protect those who are vaccinated [18]. To test the effectiveness of COVID-19 vaccines, we need to have a longer period of observation and more fully vaccinated people, as only around 35% of the older age group was vaccinated by the end of February. Second, it was not possible to distinguish between the daily cases, hospitalizations, and death rates of those who were and were not vaccinated.

### Conclusion

Vaccination in Tennessee began at the start of the “third wave,” and SARS-CoV-2–positive cases and hospitalizations had increased considerably by December and January. This work concentrated on the COVID-19 dynamics of Tennessee. The primary finding is that the vaccine should be optimally targeted at older adults as a first step, indicating that vaccination reduces daily cases for the whole population while reducing hospitalization and death rates in the older population. This study, consistent with previous studies [17], shows that mRNA COVID-19 vaccines have a protective effect for blocking transmission even after a single dose. This study also indicates that prioritizing the vaccination of the older adult population is a practical approach for reducing the number of deaths and hospitalizations.
